# Supplementary material for: Regulating photosalient behavior in dynamic metal-organic crystals
Source: Commun Chem. 2023 Jul 14;6:150. doi: 10.1038/s42004-023-00951-0 (PMC10349121; doi:10.1038/s42004-023-00951-0)
Supplement: Supplementary file 2 — Supplementary information [file 42004_2023_951_MOESM2_ESM.pdf]

Regulating photosalient behaviour in dynamic metal-organic crystals

Samim Khan,[1] Basudeb Dutta,[1] Sanobar Naaz,[1] Aditya Choudhury,[2] Pierre-Andre Cazade,[3,4] Emma Kiely,[3] Sarah Guerin[3,4]* Raghavender Medishetty,[2]*‡ and Mohammad Hedayetullah Mir[1]*†

[1]Department of Chemistry, Aliah University, New Town, Kolkata 700 156, India.

[2]Department of Chemistry, IIT Bhilai, Sejbahar, Raipur, Chhattisgarh 492015, India

[3]Department of Physics, Bernal Institute, University of Limerick, V94 T9PX, Ireland

[4]SSPC, The Science Foundation Ireland Research Centre for Pharmaceuticals, University of Limerick, V94 T9PX, Ireland

Supplementary information

Supplementary Tables

Supplementary Table 1. Crystal data and refinement parameters for compound **1**, **i1**, **p1** and **1′**.

Formula	$C_{48}H_{36}N_2O_6Zn$ **(1)**	$C_{96}H_{68}N_4O_{12}Zn_2$ **(i1)**	$C_{48}H_{36}N_2O_6Zn$ **(p1)**	$0.25(C_{96}H_{72}N_4O_{12}Zn_2)$ **(1′)**
fw	802.18	1600.28	802.18	403.83
cryst syst	Monoclinic	Monoclinic	Monoclinic	Monoclinic
space group	$P2_1/c$	$P2_1/c$	$P2_1/c$	$P2_1/c$
a (Å)	22.9098(16)	22.6733(13)	14.1626(1)	7.8757(2)
b (Å)	15.4421(10)	15.4419(4)	20.5541(1)	23.4713(8)
c (Å)	22.7056(14)	22.7005(8)	13.0975(1)	20.5487(8)
α (deg)	90	90	90	90
β (deg)	103.112(7)	103.606(4)	90.615(1)	90.977(3)
γ (deg)	90	90	90	90
V (Å^3)	7823.3(9)	7724.8(6)	3812.46(4)	3797.9(2)
T (K)	293	293	100	283
Z	8	4	4	8
D_{calcd} (g/cm^3)	1.362	1.376	1.398	1.413
μ (mm^{-1})	0.682	0.690	1.343	0.703
λ (Å)	0.71073	0.71073	0.71073	0.71073
GOF on F^2	1.024	0.940	1.031	1.47
Final R indices $[I > 2\sigma(I)]^{a,b}$	$R1$= 0.0545 $wR2$= 0.1387	$R1$= 0.0847 $wR2$= 0.2839	$R1$= 0.0285 $wR2$= 0.0774	$R1$= 0.0585 $wR2$= 0.1519

$^aR1 = \Sigma||F_o| - |F_c||/\Sigma|F_o|$, $^b\ wR2 = [\Sigma w(F_o^2 - F_c^2)^2/\Sigma w(F_o^2)^2]^{1/2}$

Supplementary Table 2. Crystal data and refinement parameters for compound **2** and **2′**.

Formula	$C_{48}H_{36}CdN_2O_6$ **(2)**	$C_{96}H_{72}Cd_2N_4O_{12}$ **(2')**
fw	849.20	1698.40
cryst syst	Monoclinic	Triclinic
space group	$P2_1/c$	$P2_1/c$
a (Å)	22.9239(9)	22.0832(11)
b (Å)	15.4394(6)	15.5975(7)
c (Å)	22.7061(9)	23.0664(10)
α(deg)	90	90
β(deg)	103.143(1)	105.172(3)
γ (deg)	90	90
V (Å^3)	7825.9(5)	7668.1(6)
Z	8	4
T (K)	293	293
D_{calcd}(g/cm^3)	1.441	1.471
μ(mm^{-1})	0.612	0.625
λ(Å)	0.71073	0.71073
GOF on F^2	1.430	1.126
Final R indices $[I > 2\sigma(I)]^{a,b}$	$R1$= 0.0969 $wR2$= 0.3622	$R1$= 0.2063 $wR2$= 0.53

$^aR1 = \Sigma||F_o| - |F_c||/ \Sigma|F_o|$, $^b wR2 = [\Sigma w(F_o^2 - F_c^2)^2/\Sigma w(F_o^2)^2]^{1/2}$

Supplementary Table 3. Selected bond lengths and bond angles **1** and **i1**.

Bond length (Å)		
	**1**	**i1**
Zn(1)-O(1)	1.955(2)	1.957(4)
Zn(1)-O(3)	1.975(2)	1.973(3)
Zn(1)-O(4)	2.566(2)	2.550(4)
Zn(1)-N(1)	2.044(2)	2.023(6)
Zn(1)-N(2)	2.038(2)	2.020(6)
Zn(2)-O(8)	2.584(2)	2.566(4)
Zn(2)-O(9)	1.951(2)	1.956(4)
Zn(2)-O(7)	1.976(2)	1.972(3)
Zn(2)-N(4)	2.032(2)	2.010(6)
Zn(2)-N(3)	2.049(2)	2.045(6)
**Bond angle (°)**		
	**1**	**i1**
O(1)-Zn(1)-O(3)	108.41(8)	109.10(15)
O(1)-Zn(1)-O(4)	163.90(8)	165.06(12)
O(1)-Zn(1)-N(1)	95.51(9)	95.1(2)
O(1)-Zn(1)-N(2)	102.23(9)	100.92(19)
O(3)-Zn(1)-O(4)	56.16(7)	56.49(13)
O(3)-Zn(1)-N(1)	113.03(9)	111.51(18)
O(3)-Zn(1)-N(2)	108.47(9)	108.7(2)
O(4)-Zn(1)-N(1)	87.74(8)	87.70(19)
O(4)-Zn(1)-N(2)	88.30(8)	88.60(18)
N(1)-Zn(1)-N(2)	126.35(9)	128.4(2)
O(7)-Zn(2)-N(4)	108.60(9)	107.4(2)
O(8)-Zn(2)-O(9)	164.83(7)	165.81(12)
O(8)-Zn(2)-N(3)	85.44(8)	85.99(19)
O(8)-Zn(2)-N(4)	88.37(8)	88.33(18)
O(9)-Zn(2)-N(3)	96.17(9)	95.2(2)

O(9)-Zn(2)-N(4)	102.60(9)	101.77(19)
N(3)-Zn(2)-N(4)	126.13(9)	128.5(2)
O(7)-Zn(2)-O(9)	110.07(8)	110.72(15)
O(7)-Zn(2)-N(3)	111.30(9)	111.20(17)
O(7)-Zn(2)-O(8)	55.79(7)	56.12(13)

Supplementary Table 4. Selected bond lengths and bond angles **1′**.

**Bond length (Å)**	
Zn(1)-O(2)	1.923(3)
Zn(1)-O(5)	1.924(3)
Zn(1)-N(3)	2.031(3)
Zn(1)-N(4)	2.010(3)
**Bond angle (°)**	
O(2)-Zn(1)-O(5)	98.41(11)
O(2)-Zn(1)-N(3)	111.16(12)
O(2)-Zn(1)-N(4)	119.58(12)
O(5)-Zn(1)-N(3)	112.58(12)
O(5)-Zn(1)-N(4)	109.04(12)
N(3)-Zn(1)-N(4)	106.10(11)

Supplementary Table 5. Selected bond lengths and bond angles **p1**.

Bond length (Å)	
Zn(1)-O(1)	1.9746(11)
Zn(1)-O(2)	2.6373(11)
Zn(1)-O(3)	2.3956(11)
Zn(1)-O(4)	2.0265(11)
Zn(1)-N(1)	2.0539(12)
Zn(1)-N(2)	2.0809(12)
**Bond angle (°)**	
O(1)-Zn(1)-O(2)	55.01(4)
O(1)-Zn(1)-O(3)	91.23(4)
O(1)-Zn(1)-O(4)	139.90(5)
O(1)-Zn(1)-N(1)	106.77(5)
O(1)-Zn(1)-N(2)	97.56(5)
O(2)-Zn(1)-O(3)	81.64(4)
O(2)-Zn(1)-O(4)	91.97(4)
O(2)-Zn(1)-N(1)	93.14(4)
O(2)-Zn(1)-N(2)	147.37(4)
O(3)-Zn(1)-O(4)	58.82(4)
O(3)-Zn(1)-N(1)	153.87(5)
O(3)-Zn(1)-N(2)	81.75(4)
O(4)-Zn(1)-N(1)	96.05(5)
O(4)-Zn(1)-N(2)	103.04(4)
N(1)-Zn(1)-N(2)	113.48(5)

Supplementary Table 6. Selected bond lengths and bond angles **2**.

**Bond length (Å)**	
Cd(1)-O(1)	1.966(3)
Cd(1)-O(2)	2.581(4)
Cd(1)-O(4)	1.940(4)
Cd(1)-N(1)	2.030(4)
Cd(1)-N(2)	2.047(4)
Cd(2)-O(10)	1.987(3)
Cd(2)-O(11)	2.557(4)
Cd(2)-O(7)	1.947(4)
Cd(2)-N(4)	2.037(4)
Cd(2)-N(3)	2.039(4)
**Bond angle (°)**	
O(1)-Cd(1)-O(2)	56.01(12)
O(1)-Cd(1)-O(4)	110.26(13)
O(1)-Cd(1)-N(1)	108.56(14)
O(1)-Cd(1)-N(2)	111.44(15)
O(1)-Cd(1)-C(35)	27.93(14)
O(2)-Cd(1)-O(4)	165.44(12)
O(2)-Cd(1)-N(1)	88.07(14)
O(2)-Cd(1)-N2	85.72(14)
O(2)-Cd(1)-C(35)	28.14(12)
O(4)-Cd(1)-N(1)	102.09(16)
O(4)-Cd(1)-N(2)	96.35(16)
O(4)-Cd(1)-C(35)	137.79(14)
N(1)-Cd(1)-N(2)	126.16(16)
N(1)-Cd(1)-C(35)	100.53(15)
N(2)-Cd(1)-C(35)	98.37(15)
O(7)-Cd(2)-O(10)	108.09(13)
O(7)-Cd(2)-O(11)	163.73(12)

O(7)-Cd(2)-N(3)	95.89(16)
O(7)-Cd(2)-N(4)	102.20(15)
O(7)-Cd(2)-C(90)	135.95(14)
O(10)-Cd(2)-O(11)	56.17(12)
O(10)-Cd(2)-N(3)	112.74(16)
O(10)-Cd(2)-N(4)	108.08(14)
O(10)-Cd(2)-C(90)	28.13(14)
O(11)-Cd(2)-N(3)	87.79(14)
O(11)-Cd(2)-N(4)	87.97(14)
O(11)-Cd(2)-C(90)	28.08(12)
N(3)-Cd(2)-N(4)	126.97(16)
N(3)-Cd(2)-C(90)	100.37(15)
N(4)-Cd(2)-C(90)	100.00(15)

Supplementary Table 7. Selected bond lengths and bond angles **2′**.

**Bond length (Å)**	
Cd(1)-O(1)	2.623(11)
Cd(1)-O(2)	1.994(10)
Cd(1)-O(4)	1.929(10)
Cd(1)-N(4)	2.042(12)
Cd(1)-N(1)a	2.060(13)
Cd(2)-O(8)	1.930(11)
Cd(2)-O(10)	1.961(11)
Cd(2)-O(11)	2.698(11)
Cd(2)-N(2)	2.039(13)
Cd(2)-N(3)	2.058(12)
**Bond angle (°)**	
O(1)-Cd(1)-O(2)	53.2(4)
O(1)-Cd(1)-O(4)	166.3(4)
O(1)-Cd(1)-N(4)	86.8(4)

O(1)-Cd(1)-C(91)	28.0(4)
O(1)-Cd(1)-N(1)a	86.3(5)
O(2)-Cd(1)-O(4)	113.8(4)
O(2)-Cd(1)-N(4)	105.1(5)
O(2)-Cd(1)-C(91)	25.2(4)
O(2)-Cd(1)-N(1)a	107.5(5)
O(4)-Cd(1)-N(4)	102.0(4)
O(4)-Cd(1)-C(91)	138.7(4)
O(4)-Cd(1)-N(1)a	95.0(5)
N(4)-Cd(1)-C(91)	98.2(5)
N(1)a-Cd(1)-N(4)	132.9(5)
N(1)a-Cd(1)-C(91)	97.0(5)
O(8)-Cd(2)-O(10)	115.2(5)
O(8)-Cd(2)-O(11)	166.5(4)
O(8)-Cd(2)-N(2)	102.3(5)
O(8)-Cd(2)-N(3)	93.5(5)
O(8)-Cd(2)-C(42)	141.0(5)
O(10)-Cd(2)-O(11)	52.3(4)
O(10)-Cd(2)-N(2)	104.6(5)
O(10)-Cd(2)-N(3)	110.5(5)
O(10)-Cd(2)-C(42)	26.1(5)
O(11)-Cd(2)-N(2)	87.1(4)
O(11)-Cd(2)-N(3)	87.5(4)
O(11)-Cd(2)-C(42)	26.2(4)
N(2)-Cd(2)-N(3)	130.2(5)

Symmetric transformation: a = 1+x, y, 1+z

Supplementary Table 8 Full DFT-calculated elastic tensors for crystals **1** and **2**. All values are in GPa.

Crystal **1** (GPa)						
	1	2	3	4	5	6
1	17.63180382	6.95569084	10.74089913	0.00000000	1.57564960	0.00000000
2	6.95569084	23.23272296	4.47885238	0.00000000	1.38414933	0.00000000
3	10.74089913	4.47885238	16.84290974	0.00000000	1.26248033	0.00000000
4	0.00000000	0.00000000	0.00000000	2.83617978	0.00000000	-0.22022507
5	1.57564960	1.38414933	1.26248033	0.00000000	7.78137203	0.00000000
6	0.00000000	0.00000000	0.00000000	-0.22022507	0.00000000	0.65661567
Crystal **2** (GPa)						
	1	2	3	4	5	6
1	14.55099899	8.37145479	8.67737470	0.00000000	1.26286940	0.00000000
2	8.37145479	17.36026458	6.76182236	0.00000000	2.76580719	0.00000000
3	8.67737470	6.76182236	18.75607341	0.00000000	2.86500950	0.00000000
4	0.00000000	0.00000000	0.00000000	2.93497408	0.00000000	0.03656777
5	1.26286940	2.76580719	2.86500950	0.00000000	7.48117757	0.00000000
6	0.00000000	0.00000000	0.00000000	0.03656777	0.00000000	0.47846697

Supplementary Figures

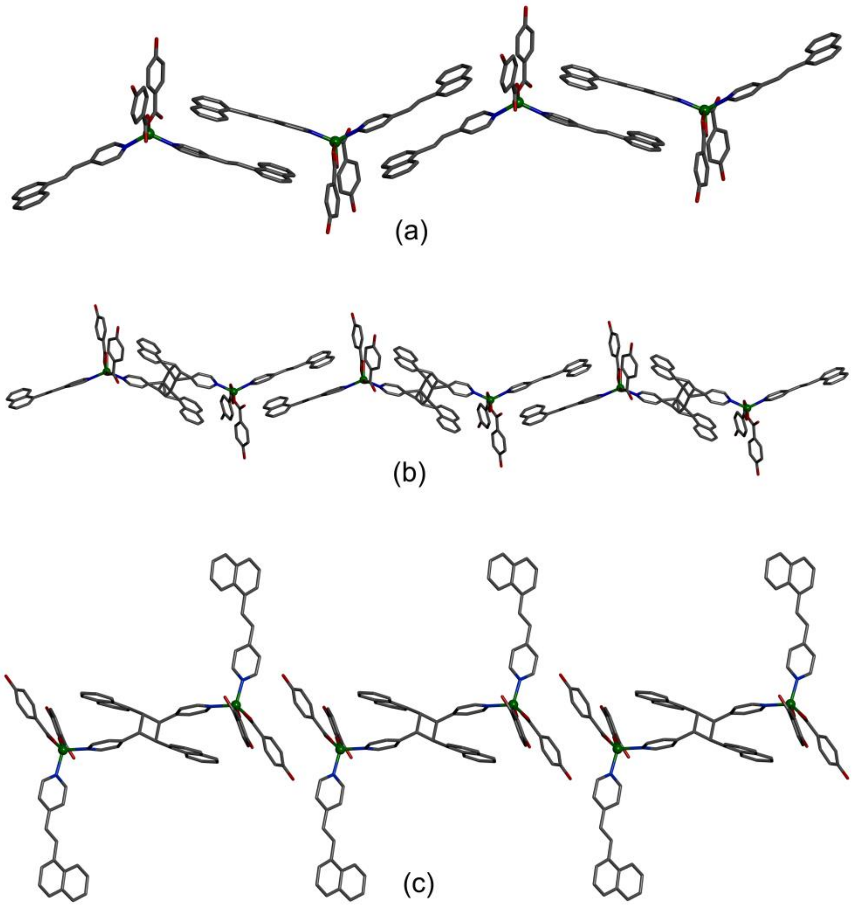

Supplementary Fig. 1: A perspective view of the compound (a) **1**; (b) **i1** and (c) **1′**.

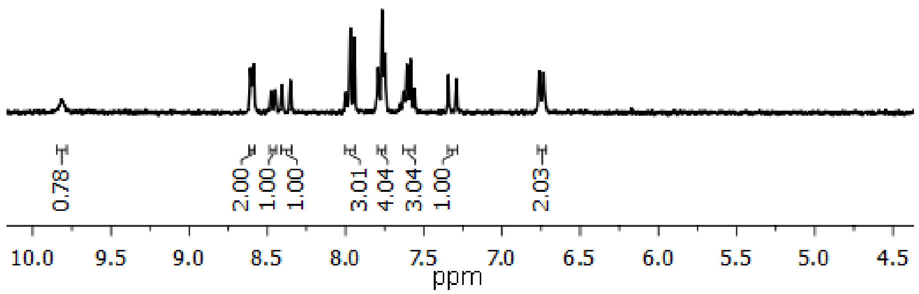

Supplementary Fig. 2: ^{1}H NMR spectrum (400 MHz, DMSO-d$_6$) of compound **1**.

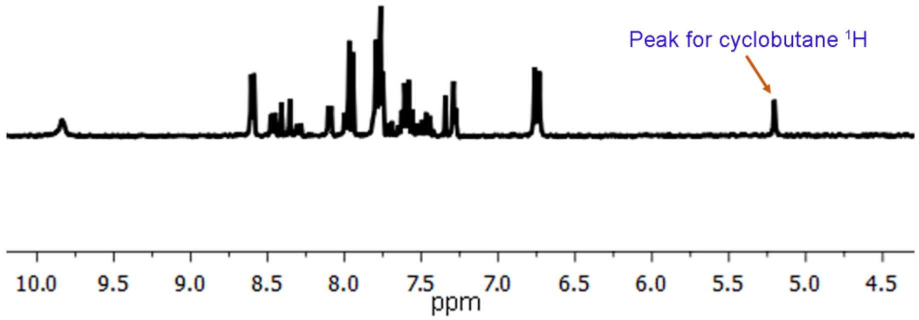

Supplementary Fig. 3: ^{1}H NMR spectrum (400 MHz, DMSO-d$_6$) of compound **1′**.

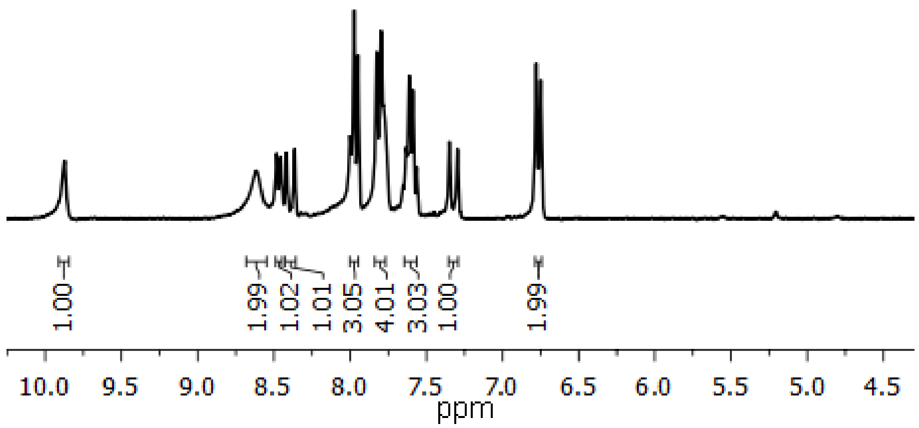

Supplementary Fig. 4: ^{1}H NMR spectrum (400 MHz, DMSO-d_6) of compound **2**.

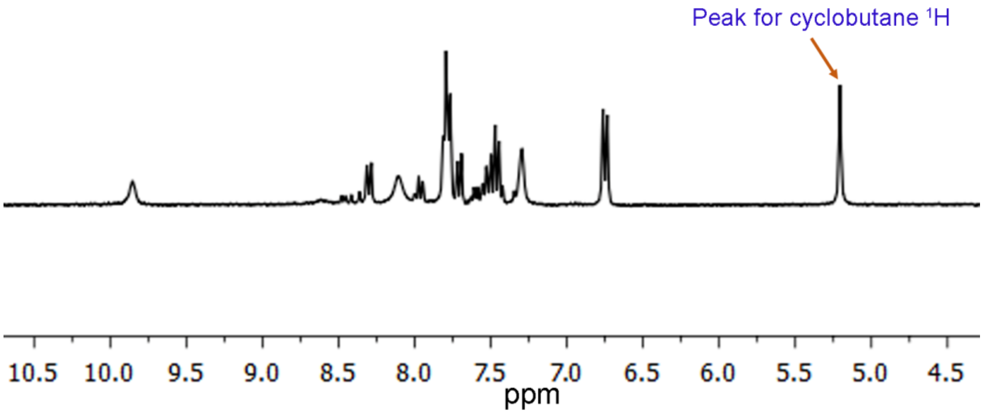

Supplementary Fig. 5: ^{1}H NMR spectrum (DMSO-d_6) of compound **2′**.

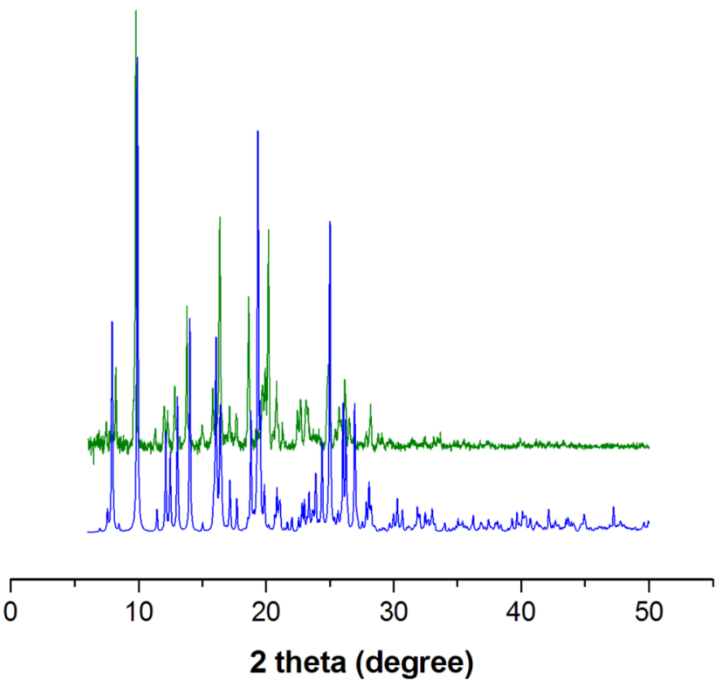

Supplementary Fig. 6: PXRD patterns of simulated (blue) and as-synthesized (olive) compound **1**.

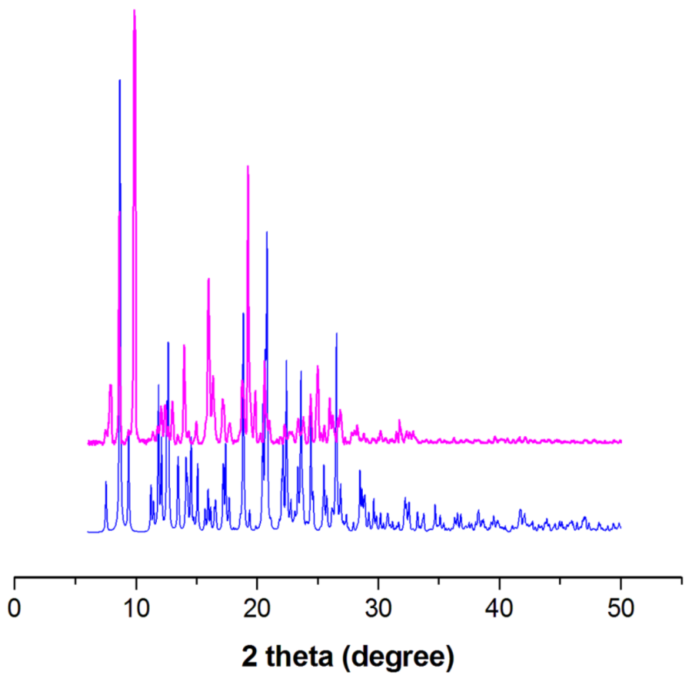

Supplementary Fig. 7: PXRD patterns of simulated (blue) and as-synthesized (magenta) compound **1′**.

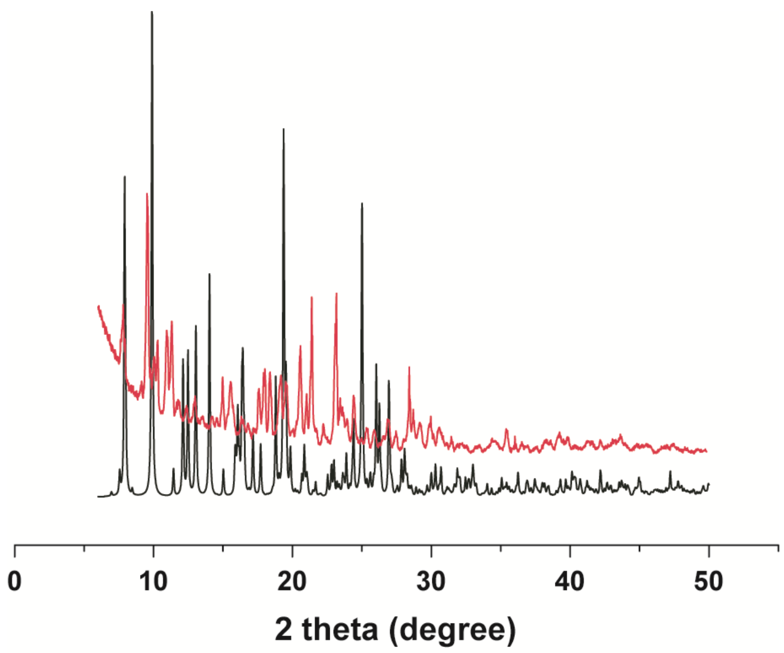

Supplementary Fig. 8: PXRD patterns of simulated (black) and as-synthesized (red) compound 2.

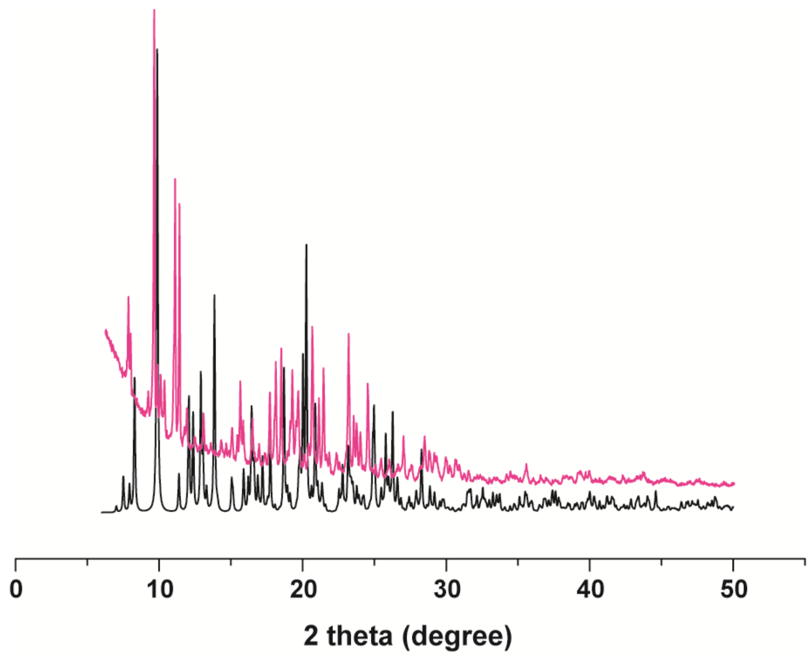

Supplementary Fig. 9: PXRD patterns of simulated (black) and as-synthesized (pink) compound 2′.

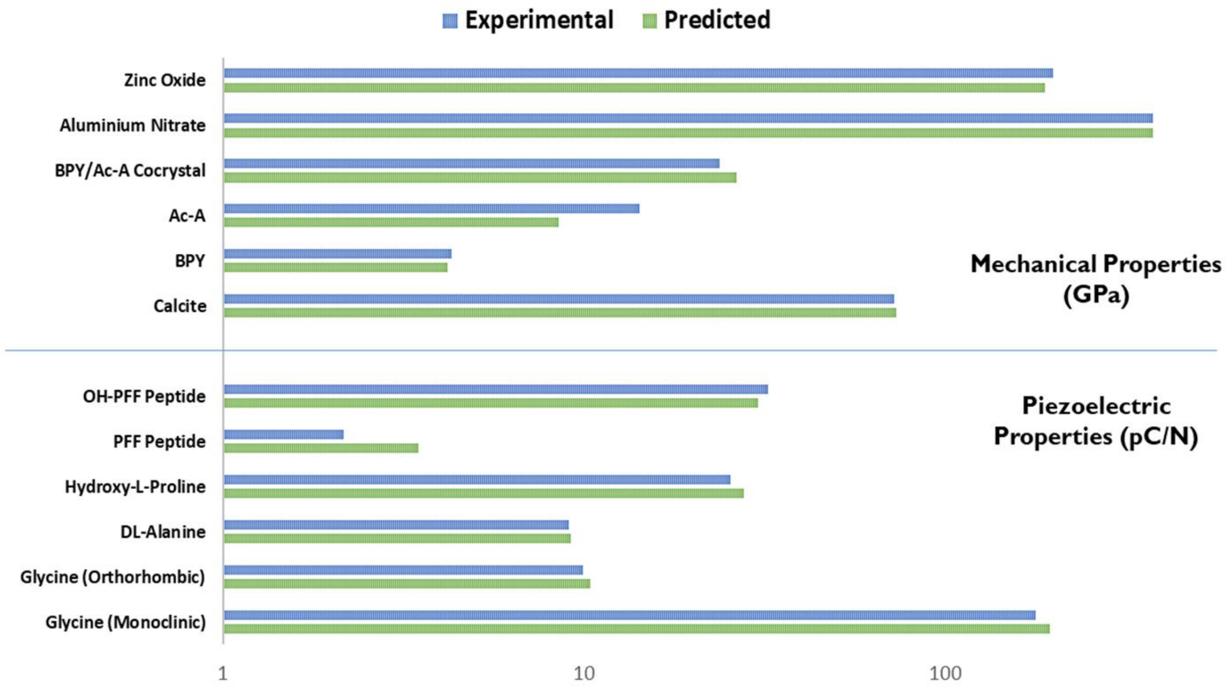

Supplementary Fig. 10: Histogram showing a snapshot of the ability of DFT calculations to predict the mechanical properties of materials across multiple crystalline material classes. Note piezoelectric properties are derived from the mechanical stiffness.

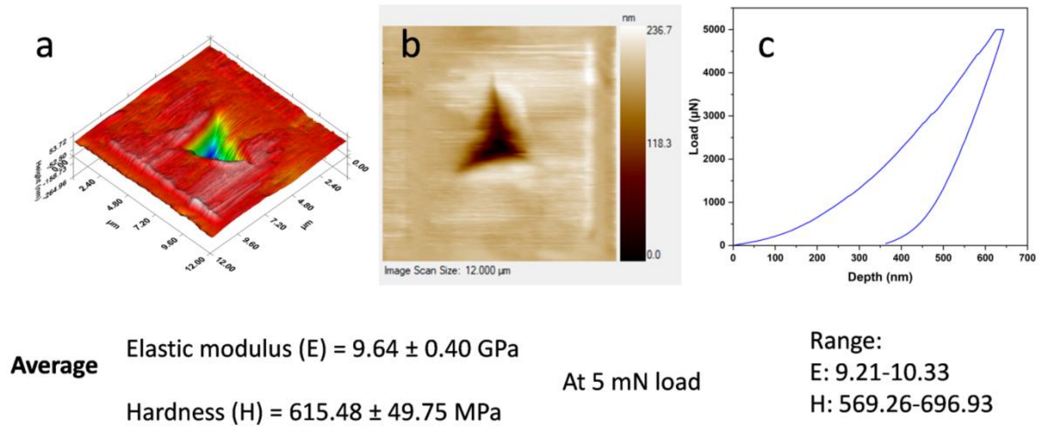

Supplementary Fig. 11: Plots and data for 5mN load for **1**. a) 3D mapped surface. b) 2D SPM image of residual indent impression, c) Load (P) – depth (h) curve.

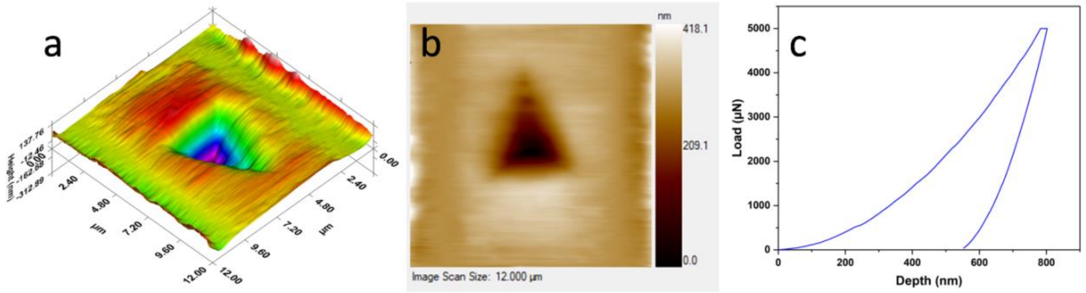

Average

Elastic modulus (E) = 9.64 ± 0.40 GPa

Hardness (H) = 615.48 ± 49.75 MPa

At 5 mN load

Range:
E: 9.21-10.33
H: 569.26-696.93

Supplementary Fig. 12: Plots and data for 5mN load for **2**. a) 3D mapped surface. b) 2D SPM image of residual indent impression. c) Load (*P*) – depth (*h*) curve.

Supplementary References

(1) G. Sheldrick, M.SHELXT–Integrated space group and crystal structure determination. *Acta Crystallogr. A*, 2015, **71**, 3–8.

(2) *SMART and SAINT*; Bruker AXS Imc.: Madison, WI, 1998.

(3) SADABS. Bruker AXS area detector scaling and absorption correction, Bruker AXS Inc., Madison, Wisconsin, USA, 2014.
