# Supplementary material for: Regulating photosalient behavior in dynamic metal-organic crystals
Source: Commun Chem. 2023 Jul 14;6:150. doi: 10.1038/s42004-023-00951-0 (PMC10349121; doi:10.1038/s42004-023-00951-0)
Supplement: Supplementary file 3 — Description of Additional Supplementary Files [file 42004_2023_951_MOESM3_ESM.pdf]

Description of Additional Supplementary Files

File name: Supplementary Data 1
Description: Details for structure 2214421

File name: Supplementary Data 2
Description: Details for structure 2214422

File name: Supplementary Data 3
Description: Details for structure 2214423

File name: Supplementary Data 4
Description: Details for structure 2214424

File name: Supplementary Data 5
Description: Details for structure 2214425

File name: Supplementary Data 6
Description: Details for structure 2214426

File name: Supplementary Movies 1-3
Description: Violent mechanical motion (jumping, splitting and bursting) of Zn crystals under UV light

File name: Supplementary Movie 4
Description: Slow mechanical motion of Zn crystal under UV light

File name: Supplementary Movie 5
Description: No mechanical motion of Zn crystal under UV light
